# Octacosanol Suppresses Lung Cancer Metastasis and Angiogenesis via Targeting MMPs and VEGF

**DOI:** 10.3390/cells15100918

**Published:** 2026-05-18

**Authors:** Mingxi Jia, Jingjing Sun, Xiuli Yang, Yue Cui, Zixuan He, Haixia Han

**Affiliations:** 1 College of Food Science and Pharmacy, Xinjiang Agricultural University, Urumqi 830052, China; 120240097@xjau.edu.cn (M.J.);; 2 Xinjiang Key Laboratory of Postharvest Fruit Science and Technology, Urumqi 830052, China; 3 College of Modern Industry in Chinese Herbal Medicine, Xinjiang Agricultural University, Urumqi 830052, China

**Keywords:** octacosanol, anti-angiogenesis, anti-metastasis, MMPs, VEGF

## Abstract

Natural bioactive compounds present promising avenues for the prevention and therapeutic intervention of cancer. Octacosanol has garnered significant attention for its distinctive biological properties, yet its specific antitumor effects and underlying mechanisms remain unclear. This study systematically evaluated its antitumor effects and elucidated the associated molecular mechanisms. We confirmed that it dose-dependently inhibited A549 cell proliferation in vitro. It also remarkably suppressed cell invasion and migration by downregulating MMP2 and MMP9 expression, an effect that was associated with reduced phosphorylation of JAK3/STAT3 and PI3K/AKT, suggesting a potential regulatory role of these signalling cascades. Meanwhile, it significantly inhibited tumor cell VEGF secretion and VEGF-mediated neoangiogenesis by modulating the PI3K/AKT signaling axis. Mouse experiments demonstrated that octacosanol significantly reduced tumor p-AKT, MMP2, and MMP9 levels, indicating its in vivo anti-metastatic effect. It also remarkably decreased tumor microvessel density, alongside reduced VEGF and vascular endothelial marker CD31 expression, further verifying its potent anti-angiogenic activity. This work provides evidence of octacosanol’s dual anti-metastatic and anti-angiogenic effects in lung cancer and offers novel mechanistic insights into its activity against this highly prevalent malignancy. These findings establish a solid foundation for further exploration and development of octacosanol as a promising adjuvant for clinical antitumor therapy.

## 1. Introduction

Cancer poses a notable threat to human health and has emerged as the leading cause of mortality in many countries worldwide, particularly those with swiftly expanding and aging populations. It is projected that by 2040, the prevalence of cancer patients will escalate to 28.4 million [1]. Hence, the development of innovative anticancer therapeutics and interventions is imperative. Natural compounds have been documented to disrupt tumor initiation, progression, and metastasis through the modulation of various pathways [2]. These natural compounds possess notable attributes such as minimal toxicity towards normal host cells and the ability to be administered over extended periods. Octacosanol, a long-chain fatty alcohol, is ubiquitously present in nature and is particularly abundant in the leaves, stems, fruits or epidermis of multiple plant species [3]. Octacosanol has a series of unique physiological functions, including anti-fatigue, anti-hypoxia, anti-oxidation, anti-inflammation, etc. [4]. Some evidence has suggested that it may also have antineoplastic influences. For instance, octacosanol derived from Tinospora cordifolia exhibits inhibitory influences on the proliferation of endothelial cells and Ehrlich ascites tumor cells. Moreover, it effectively suppressed matrix metalloproteinases 2, 9 (MMP2, MMP9), vascular endothelial growth factor (VEGF) production, and VEGF-induced neovascularization in chicken embryonic allantoic membrane and rat cornea [5]. The growth of 4T1 breast cancer cells is considerably suppressed by PEG-derived conjugates of octacosanol, with no significant impact on normal cells (HEK293 and L929) [6]. Nevertheless, there is a constrained body of research on the specific anti-tumor influences and underpinning mechanisms of octacosanol, necessitating further investigation and development of its physiological activity.

Tumors are primarily characterized by aberrant cellular proliferation and metastasis [7]. Tumor metastasis is a multifaceted phenomenon characterized by the dissemination of cancer cells from the original location to distant sites. This intricate process encompasses a series of coordinated steps, including cell proliferation, invasion, migration and angiogenesis [8,9]. These steps entail intricate interactions among various pivotal proteins and signaling pathways [10]. Among them, matrix metalloproteinases (MMPs) and VEGF are particularly linked to the progression of cancer [11]. MMPs degrade a range of extracellular matrices to enhance tumor invasion, regulate the availability of growth factors to facilitate tumor proliferation, and promote the dissemination of tumors by breaking down the vascular basement membrane and remodeling endothelial cells [12]. VEGF is mainly secreted by tumor cells and is one of the most effective pro-angiogenic factors. Hypoxia inducible factor-1 (HIF-1α) specifically targets VEGF, thereby instigating the activation of VEGFR2 and, following this step, triggering downstream signal axes that facilitate endothelial cell migration, proliferation, and tube formation [13]. The new blood vessel network provides sufficient nutrient and oxygen supply to the tumour cells, which promotes the growth and proliferation of the tumour; on the other hand, the neovascular function is initially immature and prone to leakage, which allows the cancer cells to invade the blood vessels and carry out haematogenous metastasis [14]. Consequently, targeting these factors and their upstream pathways to impede tumor metastasis and angiogenesis is regarded as a promising approach for malignancy management.

Although there have been reports revealing the anti-tumour metastatic and angiogenic potential of octacosanol, the exact mechanism remains unclear and further studies are needed to explore its efficacy across different cancer types [5,6]. As one of the most prevalent and life-threatening malignant tumors, lung cancer is associated with extremely poor prognosis, largely due to its high propensity for metastasis and aberrant angiogenesis, which are the major obstacles to effective clinical treatment. While our previous work demonstrated the dual anti-metastatic and anti-angiogenic activities of octacosanol in osteosarcoma [15], the efficacy and underlying mechanisms of this compound in lung cancer, a distinct and clinically important cancer type, remain completely unexplored. In the present study, the impact of octacosanol on the proliferation and metastasis of lung cancer cells was appraised, while its inhibitory effects on tumor angiogenesis were explored using the human umbilical vein endothelial cells (HUVECs) model. The antitumor molecular mechanisms of octacosanol were investigated using RT-qPCR and Western blot analysis. Further, murine experiments were conducted to verify its in vivo anti-metastatic and anti-angiogenic effects. The present study systematically investigates the anti-tumor mechanism of octacosanol in lung cancer, thereby extending our understanding of its broad-spectrum anti-cancer properties and providing preliminary critical preclinical evidence supporting its potential as an adjuvant therapeutic agent targeting tumor metastasis and angiogenesis for the clinical management of lung cancer.

## 2. Materials and Methods

### 2.1. Materials

Lung adenocarcinoma cell lines (A549) and HUVECs were acquired from the Affiliated Cancer Hospital of Xiangya School of Medicine (Changsha, Hunan, China). Octacosanol and cobaltous chloride (CoCl_2_) were purchased from Sigma (Burlington, MA, USA). Recombinant Human Vascular Endothelial Growth Factor 165 (VEGF165) was purchased from Sangon Biotech (Shanghai, China). SYBR qPCR Master Mix was provided by TransGen Biotech (Beijing, China). Antibodies against PI3K (cat: 4257T), p-PI3K (cat: 4228T), AKT (cat: 9272S), p-AKT (cat: 9271T), and GAPDH (cat: 5174S) were acquired from Cell Signal Technology (Boston, MA, USA). Antibodies against HIF-1α (cat: AF1009), MMP2 (cat: AF5330) and MMP9 (cat: AF5228) were provided by Affinity Biosciences (Cincinnati, OH, USA). Antibodies for JAK3 (cat: AB45141), p-JAK3 (cat: AB278789), and STAT3 (cat: AB76315) were sourced from Abcam (Cambridge, UK).

### 2.2. Preparation of Octacosanol Nanoemulsion

The high hydrophobicity of octacosanol significantly impedes its solubility and bioavailability, thereby impacting its efficacy. To address these challenges, we formulated octacosanol nanoemulsions based on previous research findings [16]. In brief, octacosanol (0.2 g) was dissolved and dispersed in ethyl acetate (2 mL) at 85 °C. Subsequently, PEG40 hydrogenated castor oil (1.2 mL) was added to the system and thoroughly stirred. Further addition of deionized water (14.8 mL) took place while maintaining continuous heating and stirring for 5 min. The octacosanol nanoemulsion was obtained by rapidly cooling the mixture in water at 25 °C. Additionally, a blank emulsion was prepared using equal amounts of ethyl acetate and PEG40-hydrogenated castor oil. All prepared emulsions were stored at 4 °C in the present study, and our previous systematic validation has confirmed that this nanoemulsion system can remain stable for at least 60 days under 4 °C storage, as well as exhibiting excellent tolerance to repeated freeze–thaw cycles [16]. For cell-based assays, the octacosanol nanoemulsion was directly diluted into the culture medium to achieve the desired final concentrations (maximum concentration: 120 μg/mL). Based on the nanoemulsion preparation, the final concentrations of ethyl acetate and PEG40 hydrogenated castor oil in the culture medium ranged from 0.08% to 0.12% (*v*/*v*) and 0.05% to 0.07% (*v*/*v*), respectively. These solvent concentrations are well below the levels typically associated with cytotoxicity.

### 2.3. Cell Culture

A549 cells and HUVECs were cultured in Dulbecco’s Modified Eagle’s Medium (DMEM, high glucose, with L-glutamine, Gibco, CA, USA) supplemented with 10% fetal bovine serum (FBS, Gibco, CA, USA) and 1% penicillin-streptomycin (100 U/mL penicillin and 100 μg/mL streptomycin, Solarbio, BeiJing, China). The medium contained phenol red (standard DMEM formulation). Cells were maintained at 37 °C in a humidified atmosphere containing 5% CO_2_. For serum-free treatments (e.g., in transwell migration assays, gelatin zymography, and tube formation assays), cells were washed twice with phosphate-buffered saline (PBS) and then incubated in DMEM without FBS (still containing phenol red) for the indicated durations. These same culture conditions were used in all subsequent in vitro experiments.

### 2.4. Cell Viability Assay

The cells were inoculated in a 96-well plate and exposed to octacosanol for 24 h, followed by the addition of CCK8 working solution to each well. After incubation for 2 h, the absorbance at 450 nm was measured using a microplate reader.

### 2.5. Cell Migration by the Transwell Assay

Dispersed 300 µL of cells (1 × 10^5^ cells/mL) in serum-free medium containing varying concentrations of octacosanol, and subsequently inoculated them into 24-transwell cell culture chambers. Added 700 µL of medium supplemented with 20% fetal bovine serum (FBS) to the basolateral side. Unmigrated cells on the upper surface of the chamber membrane were gently wiped off using a cotton swab after 12 h of incubation. The migrated cells on the lower surface were fixed with 4% paraformaldehyde for a duration of 15 min and subsequently stained with 0.5% crystal violet for a period of 20 min. The migratory cells were then observed and captured using an inverted microscope.

### 2.6. 3D Trials of Cell Invasion

The effect of octacosanol on the invasive ability of A549 cells was assessed following the methodology described by Berens et al. [17]. In short, 30 sets of A549 single-cell suspension (1 × 10^5^ cells/ mL) were inoculated onto the lid of a 10 cm dish, with each set comprising 20 μL, and incubated over a 72 h period to facilitate the formation of cellular aggregates. Following this step, 300 μL of cell clusters were gently mixed with 80 μL of Matrigel at a temperature of 4 °C. The mixture was inoculated into a 24-well plate at a volume of 30 μL per well and incubated for 40 min to allow solidification. Thereafter, complete medium supplemented with octacosanol was added. The infiltrative potential of the cell clusters in the surrounding area was noted and logged under a microscope within 48 h.

### 2.7. Gelatin Zymography

A549 cells were seeded into six-well plates and cultured overnight, followed by replacement with serum-free medium containing various concentrations of octacosanol. After 24 h, conditioned media were collected and normalized for protein content. Equal amounts of protein were separated on 8% SDS-polyacrylamide gels containing 1 mg/mL gelatin. Following electrophoresis, gels were washed in 2.5% Triton X-100 to remove SDS and incubated in developing buffer at 37 °C for 48 h. Gels were then stained with Coomassie Brilliant Blue R-250, and gelatinolytic activity was visualized as clear bands against a blue background. The activities of MMP2 and MMP9 were quantified using ImageJ 1.8.0 software (National Institutes of Health, Bethesda, MD, USA) [18].

### 2.8. Western Blot

A549 cells were seeded into six-well plates and treated with octacosanol for 24 h. Total protein was extracted using RIPA buffer containing protease and phosphatase inhibitors, and protein concentrations were determined by BCA assay. Equal amounts of protein were separated by SDS-PAGE and transferred onto PVDF membranes. After blocking with 5% non-fat milk, membranes were incubated overnight at 4 °C with primary antibodies, followed by HRP-conjugated secondary antibodies. Protein bands were visualized using an enhanced chemiluminescence system (Bio-Rad, Hercules, CA, USA) and quantified with ImageJ software, with GAPDH used as the loading control [15].

### 2.9. RT-qPCR

Total RNA was extracted from treated cells using a spin column-based kit. Specifically, the TransZol Up Plus RNA Kit (ER501, TransGen Biotech, Beijing, China) was employed following the manufacturer’s protocol, which combines the strong lysis capacity of TransZol Up with centrifugal column purification to efficiently isolate high-purity total RNA without the need for chloroform extraction. Reverse transcription was carried out with the HiScript III All-in-one RT SuperMix (Vazyme, Nanjing, China) following the manufacturer’s instructions. Quantitative PCR was performed using the Universal SYBR Green Fast qPCR Mix (Abbkine, Wuhan, China) on a real-time PCR system. The primer sequences used for amplification are listed in Table 1. GAPDH served as the internal reference gene, and relative gene expression levels were calculated using the 2^−ΔΔCt^ method.

### 2.10. HUVECs Tube Generation

Matrigel (40 μL) was evenly spread into each well of a pre-chilled 96-well plate and incubated at 37 °C for 30 min to allow polymerization. HUVECs were harvested, resuspended in medium containing various concentrations of octacosanol, and seeded into the Matrigel-coated wells at a density of 2 × 10^5^ cells per well (200 μL total volume). After 3 h of incubation at 37 °C in a 5% CO_2_ atmosphere, tube structures were visualized under an inverted microscope. Five random fields per well were photographed, and the number of branch points or total tube length was quantified using ImageJ software to assess angiogenic capacity.

### 2.11. Chick Embryo Chorioallantoic Membrane (CAM) Angiogenesis

The freshly fertilized chick embryos were incubated in a humidified incubator set at a temperature of 37 ± 0.5 °C. On the eighth day of embryonic development, a rectangular aperture was created on the eggshell. A specific region on the CAM was selected and designated, followed by the application of VEGF165 or octacosanol. Subsequently, the aperture was sealed using sterile parafilm. Each experimental group consisted of five eggs. After 48 h, the CAM was fixed for 15 min using a fixative solution composed of ethanol and acetone in a ratio of 1:1. Subsequently, the CAM was excised and placed on filter paper to facilitate observation of microvessel density changes, followed by capturing images.

### 2.12. Transplanted Tumour Models and In Vivo Trials

Laboratory animals were housed in accordance with the Chinese Guidelines for the Care and Use of Laboratory Animals. The animal study protocol was approved by the Ethics Committee of Xinjiang Agricultural University Laboratory Animal Centre (approval No. 2024107). We conducted murine experiments using a subcutaneous (flank) xenograft tumor model, which is the most widely used standard model in preclinical drug development for initial assessment of compound efficacy. This model offers the advantages of technical simplicity, a high tumor take rate, consistent tumor growth kinetics, and convenient monitoring of tumor progression, making it ideal for evaluating octacosanol’s anti-tumor, anti-metastatic, and anti-angiogenic activities in the context of lung cancer and laying a solid foundation for future investigations using more complex orthotopic models. Specifically, four-week-old male BALB/c nude mice were housed in groups under specific pathogen-free conditions, and all surgeries were performed under aseptic conditions. A549 cells (3 × 10^7^ cells/mL) were injected into the right superolateral subcutaneous tissue of nude mice. The mice were grouped into 6 mice per group. The mice in the experimental group were gavaged with 30 mg/kg octacosanol nanomedicine working solution every two days; the negative control (NC) group was gavaged with an equal amount of blank emulsion, and the blank control group was treated with an equal amount of PBS solution. The length (L) and width (W) of the tumours were measured every three days and the tumour volume was calculated using the formula (L × W^2^)/2. After 30 days of drug administration, the animals were executed and the tumours were harvested and weighed.

### 2.13. Pathological and Immunohistochemical Assays

Tissue samples were dehydrated and embedded in paraffin, sectioned at a thickness of 5 μM, stained with standard hematoxylin-eosin (H&E) staining for histopathological analysis, and protein expression levels in the tumour samples were assessed by immunohistochemistry (IHC) assay. Serial sections were also stained with PBS instead of the primary antibody as a control.

### 2.14. Statistical Analysis

All experiments were performed independently at least three times, and the data are expressed as the mean ± standard deviation (SD). Statistical comparisons between two groups were conducted using Student’s *t*-test, while comparisons involving more than two groups were analyzed by one-way analysis of variance (ANOVA) followed by Tukey’s post hoc test. Analyses were carried out using SPSS version 20.0 (IBM Corp., Armonk, NY, USA) or GraphPad Prism version 8.0 (San Diego, CA, USA). A value of *p* < 0.05 was considered to indicate a statistically significant difference.

## 3. Results

### 3.1. Characterisation of Octacosanol Nanoemulsions and Effect on A549 Cell Viability

**Figure 1:**
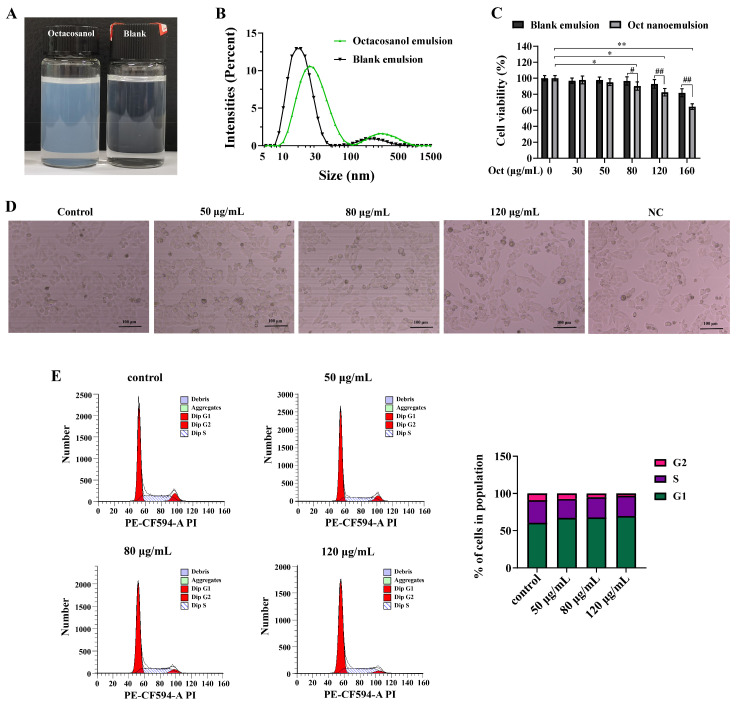
Appearance and cytotoxicity of octacosanol nanoemulsions. (**A**) Appearance of octacosanol nanoemulsion. (**B**) Particle size distribution of nanoemulsions. (**C**) Cell viability was assessed by CCK-8 assay. (**D**) Morphology of A549 cells after exposure to octacosanol nanoemulsion. A blank emulsion equal to the highest concentration group of octacosanol nanoemulsion was used as a negative control (NC). (**E**) Cell cycle analysis. All data represent mean ± SD of *n* = 3 biological replicates (three independent experiments), with technical triplicates in each experiment. Comparisons between two groups were performed using Student’s *t*-test. # *p* < 0.05 and ## *p* < 0.01 vs. blank emulsion group; * *p* < 0.05 and ** *p* < 0.01 vs. control.

The prepared octacosanol nanoemulsion was clear and transparent, with an average particle size of about 30 nm and uniform distribution of droplets (Figure 1A,B). The system effectively improved the solubility of octacosanol. When the concentration of octacosanol ranged from 80 to 120 μg/mL, it exhibited inhibitory effects on the proliferation of A549 cells (Figure 1C). However, at a concentration of 160 μg/mL, even the equivalent blank emulsion demonstrated cytotoxicity. To minimize solvent interference, the octacosanol emulsion dosage should not exceed 120 μg/mL. Consequently, these doses were chosen for further investigation. It is important to note that the blank emulsion alone showed no cytotoxic effect at concentrations corresponding to ≤120 μg/mL octacosanol, confirming that the biological activities observed in this study are specifically mediated by octacosanol, not by the formulation excipients. Consequently, these doses were chosen for further investigation. After 24 h of octacosanol treatment, no significant alterations in cell morphology were observed, and apoptosis levels remained statistically insignificant (Figure 1D). Meanwhile, as the concentrations of octacosanol increased, the A549 cell cycle exhibited an accumulation in the G1 phase and a decrease in the S and G2 phases (Figure 1E). These findings suggested that octacosanol may exert antitumor effects through its inhibitory effect on cell proliferation.

### 3.2. Octacosanol Inhibited the Invasion and Migration of A549 Cells

**Figure 2:**
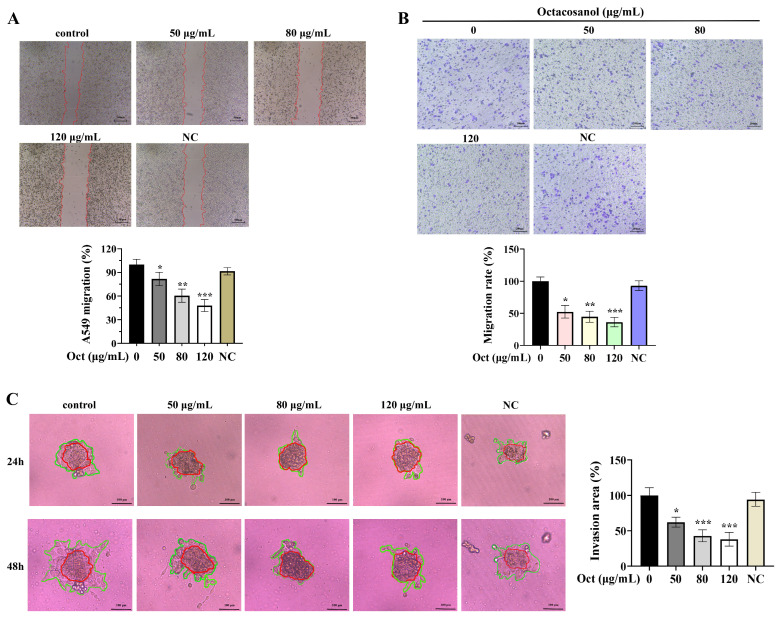
Octacosanol inhibits the ability of A549 cells to invade and migrate. (**A**) Scratches were made by scraping confluent cell monolayers, and wound areas were photographed and counted 24 h later (scale bar: 500 μm). The red line indicates the boundary of the wound healing process in the cell. (**B**) The effect of octacosanol on cell migration was evaluated by transwell assay. Photographs and recordings were taken of five randomly selected fields of view in each group, followed by the counting and averaging of the quantity of migrated cells (scale bar: 200 μm). (**C**) Inhibitory effect of octacosanol on A549 cell invasion in 3D model. The red part represented the original cell cluster, and the green part was the outward invasion area (scale bar: 100 μm). The blank emulsion was the negative control (NC). All data represent mean ± SD of *n* = 3 biological replicates (three independent experiments), with technical triplicates in each experiment. Comparisons between two groups were performed using Student’s *t*-test. * *p* < 0.05, ** *p* < 0.01, *** *p* < 0.001 vs. control.

The inhibitory effect of octacosanol on cell migration was analyzed through wound healing and transwell assays. As shown in Figure 2, octacosanol treatment significantly reduced A549 cell migration in a dose-dependent manner. Meanwhile, the transwell migration assay also confirmed that octacosanol inhibited the migration of cancer cells. The 3D experiments of cell invasion suggested that the invasion ability of A549 cells was weakened by octacosanol in a dose-dependent manner. The area ratio of cell invasion was reduced by approximately 37.8–61.9% compared to the control group. These results suggested that octacosanol effectively inhibited the migration and invasion ability of A549 cells.

### 3.3. Octacosanol Inhibits MMP Expression

**Figure 3:**
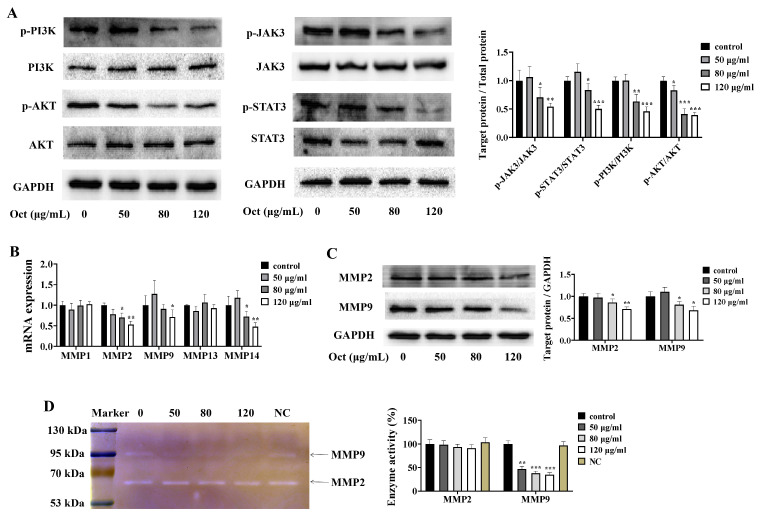
Signaling pathways associated with tumor cell metastasis. (**A**) Octacosanol inhibits the phosphorylation-mediated activation of the JAK3/STAT3 and PI3K/AKT pathways. (**B**) RT-qPCR was employed to detect the mRNA levels of MMPs in the cells, with GAPDH serving as the internal reference gene. (**C**) Gelatin zymography was utilized to evaluate the activity of MMP2 and MMP9. (**D**) Protein levels of MMP2 and MMP9 in cell lysates were measured through Western blot. The intensity of the bands was quantified by brightness analysis using ImageJ software. All data represent mean ± SD of *n* = 3 biological replicates (three independent experiments), with technical triplicates in each experiment. Comparisons between two groups were performed using Student’s *t*-test. * *p* < 0.05, ** *p* < 0.01, *** *p* < 0.001 vs. untreated control.

To evaluate the antitumor effects of octacosanol, we examined the expression of genes associated with cell invasion and found that octacosanol treatment led to reduced phosphorylation levels of key components of the JAK3/STAT3 and PI3K/AKT pathways in a dose-dependent manner, indicating a correlation between octacosanol exposure and decreased activation of these pathways (Figure 3A). It is noteworthy that matrix metalloproteinases (MMPs), downstream genes regulated by the JAK3/STAT3 and PI3K/AKT pathways, play a pivotal role in facilitating tumor invasion and migration through the degradation of extracellular matrix (ECM) components. To investigate the inhibitory impact of octacosanol on MMPs, the mRNA expression levels of MMP-1/2/9/13/14 were assessed using RT-qPCR, while the protein levels and proteolytic activity of MMP2 and MMP9 were determined through Western blotting and gelatin zymography. Treatment with octacosanol significantly suppressed the levels of MMP-2/9/14, as demonstrated in Figure 3B. However, there were no significant changes observed in the mRNA expression of MMP1 and MMP13. In enzymatic assays conducted on A549 cells (Figure 3C), octacosanol treatment effectively inhibited the proteolytic activity of MMP9, while only a marginal decrease was observed in the level of MMP2. The findings presented in Figure 3D suggested a significant reduction in the expression levels of MMP2 and MMP9 proteins in a dose-dependent manner upon treatment with octacosanol. These results suggested that the anti-tumor metastatic effects of octacosanol were closely related to their targeted modulation of MMPs, especially MMP2 and MMP9.

### 3.4. Octacosanol Reverses Hypoxia-Induced Upregulation of Pro-Metastatic Proteins in A549 Cells

**Figure 4:**
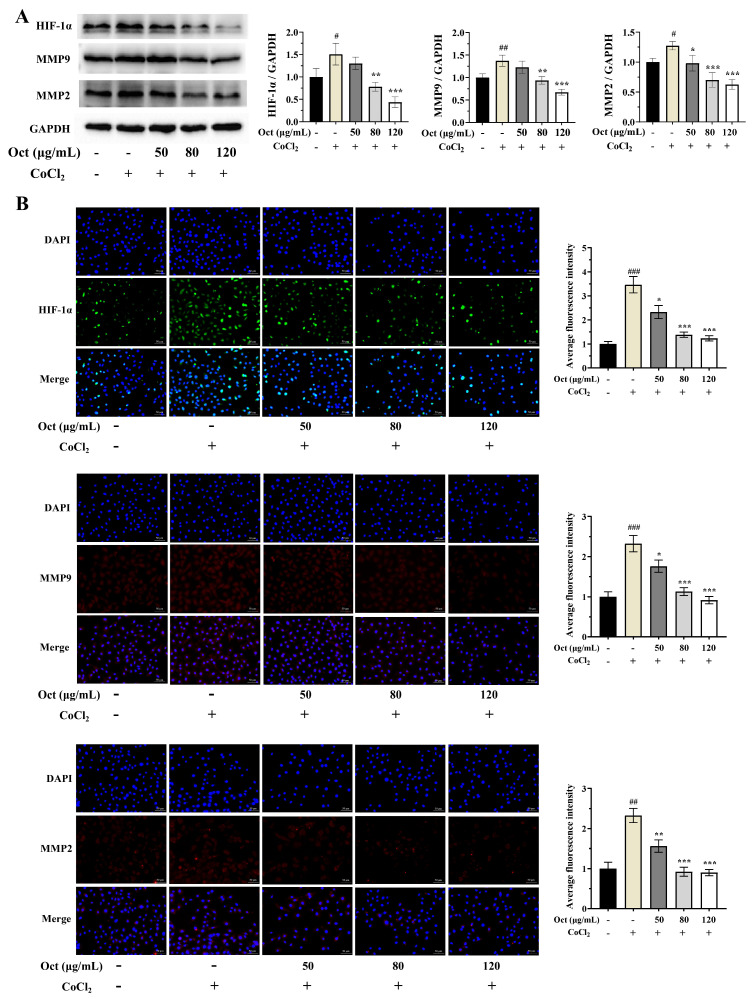
Octacosanol inhibited the upregulation of HIF-1α, MMP2, and MMP9 in A549 cells under hypoxic conditions in a dose-dependent manner. (**A**) Representative Western blot results. (**B**) Representative immunofluorescence staining results (scale bar: 50 μm; original magnification: 200×). All data represent mean ± SD of *n* = 3 biological replicates (three independent experiments), with technical triplicates in each experiment. Comparisons between two groups were performed using Student’s *t*-test. # *p* < 0.05, ## *p* < 0.01, ### *p* < 0.001 vs. blank control; * *p* < 0.05, ** *p* < 0.01, *** *p* < 0.001 vs. positive control.

To investigate the regulatory role of octacosanol on hypoxia-associated metastatic phenotypes in A549 cells, we established a chemical hypoxia model by treating these cells with CoCl_2_. We then assessed the protein expression levels of HIF-1α, MMP2 and MMP9, well-established key mediators of tumor invasion and metastasis under hypoxic microenvironments. The results demonstrated that compared with the normoxic control group, CoCl_2_-induced hypoxia significantly upregulated the protein expression of HIF-1α, MMP2, and MMP9 (Figure 4). This observation not only confirmed the successful establishment of our hypoxic cell model but also verified the activation of the downstream pro-metastatic signaling cascade triggered by hypoxic stress. Notably, octacosanol treatment markedly reversed this hypoxia-induced aberrant upregulation of these proteins in a dose-dependent manner. With increasing octacosanol concentrations, the protein levels of HIF-1α, MMP2, and MMP9 were gradually and significantly attenuated, a trend consistently supported by both quantitative analysis of Western blot bands and semi-quantitative assessment of immunofluorescence staining intensity.

### 3.5. Octacosanol Exerts Anti-Tumor Effects via Suppressing Hypoxia-Driven PI3K/AKT and Pro-Angiogenic Signaling

**Figure 5:**
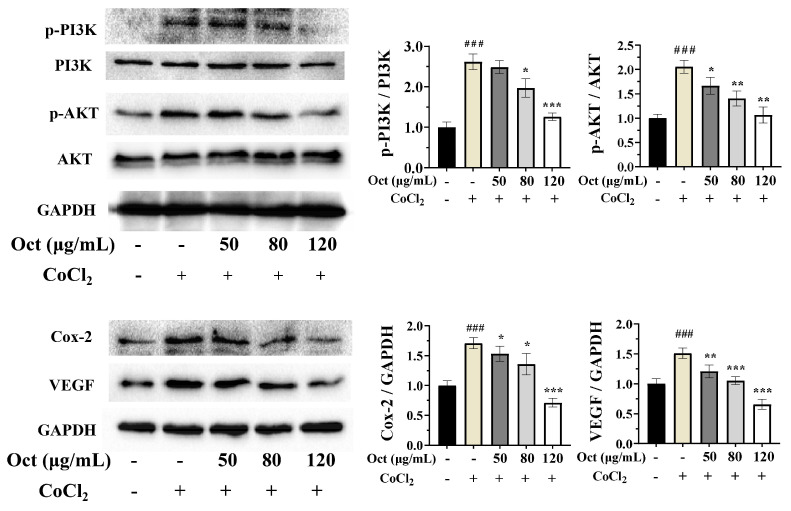
Octacosanol inhibits the activation of the PI3K/AKT pathway under hypoxic conditions, as well as the expression of Cox-2 and VEGF. All data represent mean ± SD of *n* = 3 biological replicates (three independent experiments), with technical triplicates in each experiment. Comparisons between two groups were performed using Student’s *t*-test. ### *p* < 0.001 vs. blank control; * *p* < 0.05, ** *p* < 0.01, *** *p* < 0.001 vs. positive control.

To further explore the underlying molecular mechanism of this inhibitory effect, we next examined the activation status of the canonical upstream signaling pathway that regulates HIF-1α stabilization under hypoxia, namely the PI3K/AKT cascade (Figure 5). As expected, CoCl_2_-induced hypoxia markedly induced the phosphorylation of PI3K and AKT, indicating robust activation of this pathway in response to hypoxic stress. Consistent with our earlier findings, octacosanol treatment significantly suppressed this hypoxia-induced phosphorylation of PI3K and AKT in a dose-dependent manner. Vascular endothelial growth factor (VEGF) expression is regulated by HIF-1α, and elevated levels of HIF-1α promote increased VEGF expression, thereby playing a crucial role in angiogenesis. The findings suggested that octacosanol could effectively suppress the activation of the PI3K/AKT pathway and the ERK pathway, thereby inhibiting HIF-1α accumulation and VEGF expression under hypoxic conditions (Figure 5). Cyclooxygenase-2 (Cox-2) has the ability to induce the expression of pro-angiogenic factors, such as VEGF and bFGF, thereby facilitating tumor angiogenesis. Under hypoxic conditions, octacosanol effectively suppressed the expression of Cox-2 protein.

Collectively, these findings indicated that octacosanol effectively blocked hypoxia-induced activation of PI3K/AKT signaling, thereby attenuating downstream upregulation of HIF-1α and its target MMPs, ultimately suppressing the invasive and metastatic potential of hypoxic tumor cells. The findings further underscored the inhibitory potential of octacosanol against invasion, metastasis and aberrant angiogenesis in tumors.

### 3.6. Octacosanol Inhibited HUVECs Tube Formation and CAM Microvessel Density

**Figure 6:**
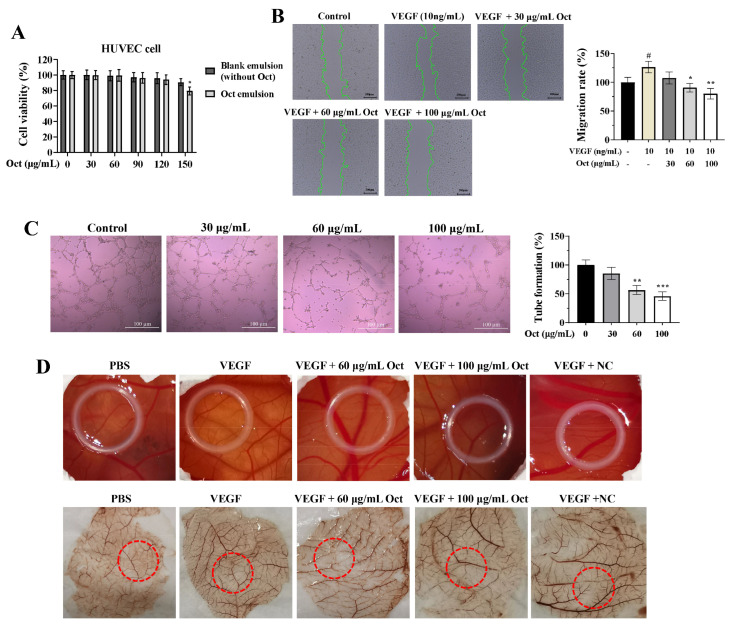
Antiangiogenic effects of octacosanol under nontoxic conditions. (**A**) Viability of HUVECs assessed by CCK-8 assay. (**B**) Wound healing assay showing octacosanol inhibition of VEGF-induced HUVECs migration (blank control: untreated; positive control: VEGF). Scale bar: 200 μm. # *p* < 0.05 vs. control group; * *p* < 0.05, ** *p* < 0.01 vs. VEGF group. (**C**) Tube formation assay on Matrigel after 3 h of culture. Scale bar: 100 μm. ** *p* < 0.01, *** *p* < 0.001 vs. control. (**D**) CAM assay showing neovascularization changes 48 h after treatment; circles indicate application areas. Blank emulsion served as negative control (NC). All data represent mean ± SD of *n* = 3 biological replicates (three independent experiments), with technical triplicates in each experiment.

HUVECs were utilized as an in vitro model system to investigate the progression of angiogenesis. Octacosanol exhibited negligible cytotoxicity on HUVECs at doses below 120 μg/mL (Figure 6A). Wound healing experiments demonstrated that octacosanol significantly impeded VEGF-induced migration of HUVECs (Figure 6B). Figure 6C indicates that octacosanol significantly inhibited the formation of tube-like structures in HUVECs. In the CAM model, vascular regions were induced by VEGF during embryonic development. Notably, octacosanol demonstrated a concentration-dependent inhibition of neovascularization, highlighting its potential as an anti-angiogenic agent (Figure 6D).

### 3.7. Octacosanol Inhibited VEGF-Induced Activation of Phosphorylation of VEGFR2 and Downstream Signaling Pathways

**Figure 7:**
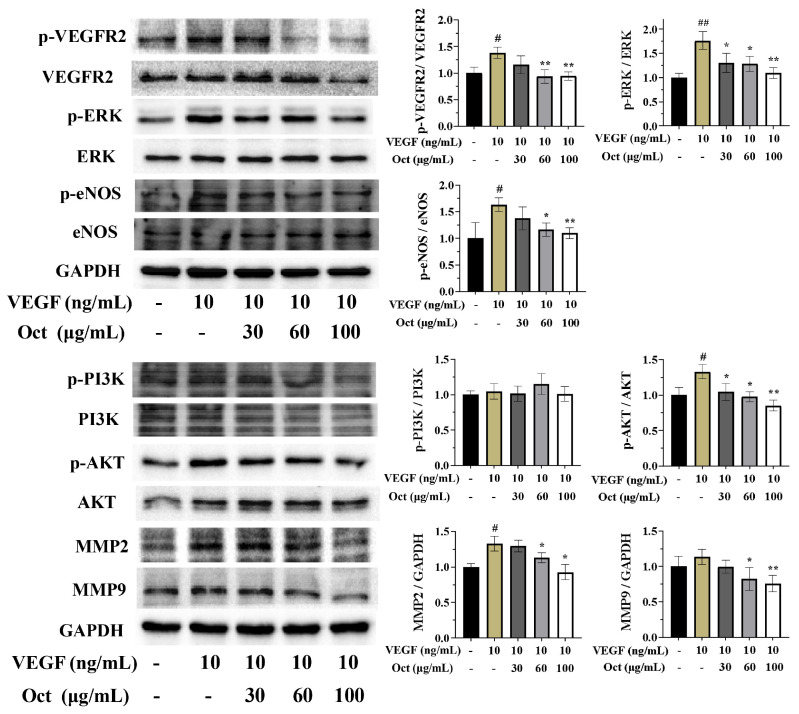
Octacosanol effectively inhibited VEGF-induced phosphorylation activation of VEGFR2 and downstream signaling pathways. The untreated group was the blank control, and the VEGF group was the positive control. All data represent mean ± SD of *n* = 3 biological replicates (three independent experiments), with technical triplicates in each experiment. Comparisons between two groups were performed using Student’s *t*-test. # *p* < 0.05 and ## *p* < 0.01 vs. positive control; * *p* < 0.05 and ** *p* < 0.01 vs. blank control.

VEGFR2 serves as a crucial signaling molecule for angiogenesis and endothelial cell mitosis. As depicted in Figure 7, octacosanol treatment resulted in the down-regulation of p-VEGFR2 in VEGF-stimulated HUVECs, while the overall expression level of VEGFR2 protein remained unchanged. To elucidate the anti-angiogenic mechanism of octacosanol in HUVECs, an analysis was conducted on several key kinases involved in the VEGFR2-mediated angiogenic pathway. Figure 7 suggested that octacosanol significantly inhibited VEGF-induced phosphorylation of AKT, ERK and eNOS in HUVECs. Interestingly, octacosanol decreased p-PI3K and total PI3K protein levels, but no significant changes in p-PI3K/PI3K levels. Furthermore, octacosanol dose-dependently suppressed the expression of MMP2 and MMP9. In aggregate, our findings suggested that octacosanol possesses the ability to inhibit VEGF-induced activation of VEGFR2 and selectively target downstream signal transduction events mediated by VEGFR2, thereby exerting its anti-angiogenic effect.

### 3.8. Octacosanol Inhibits Tumour Cell Migration and Angiogenesis In Vivo

**Figure 8:**
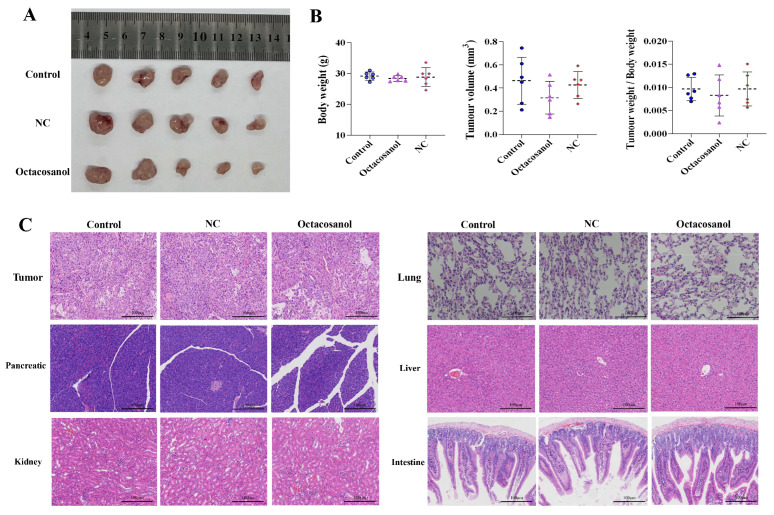
In vivo antitumour effects of octacosanol. (**A**) Tumour mass appearance. (**B**) Mouse body weight and tumour weight. (**C**) H&E staining of tumour and lung tissues. Scale bar = 100 μm (applies to all panels).

A mouse subcutaneous tumour model was established to further investigate the in vivo tumour inhibitory function of octacosanol. The blank control mice were gavaged with PBS, and the negative control (NC) group was gavaged with an equal amount of blank emulsion. Figure 8 showed that the tumour growth rate was inhibited and the tumour mass size was relatively smaller in the octacosanol-treated group compared with the control group, but this difference did not reach statistical significance (*p* > 0.05). This lack of significance is likely attributable to the relatively small sample size (*n* = 6 per group) coupled with high inter-individual variability in the growth kinetics of the xenograft tumors, which is commonly encountered in this preliminary preclinical evaluation. In addition, in vivo weighing of mice at the end of the test showed that octacosanol had no significant effect on the body weight of mice. H&E staining in Figure 8C suggested that the tumour tissue had a high cell density, disordered arrangement and a large proportion of nuclear schizophrenic cells. Compared to the control group, there was no significant difference in the morphology, density, necrosis and proliferative capacity of the tumour cells by octacosanol, whereas it significantly inhibited the microvessel density in the tumour tissues. Meanwhile, histopathological examination of the lung, liver, kidney and small intestine of mice showed that the dose of octacosanol used had no toxic side effects and had a good safety profile (Figure 8C).

**Figure 9:**
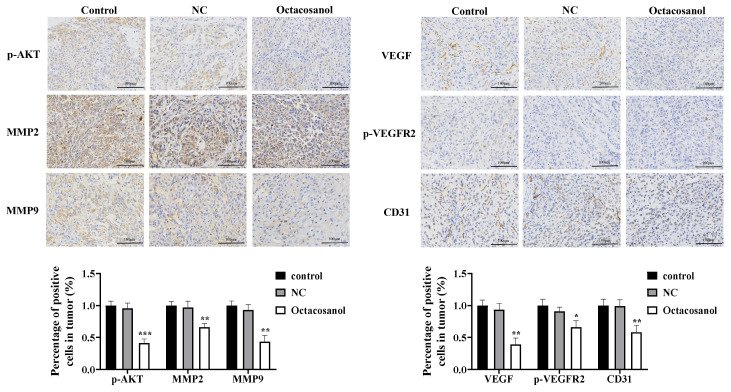
Immunohistochemical detection of p-AKT, MMP2, MMP9, VEGF, p-VEGFR2 and CD31 in tumour tissues. All data represent mean ± SD of *n* = 3 biological replicates (three independent experiments), with technical triplicates in each experiment. Comparisons between two groups were performed using Student’s *t*-test. * *p* < 0.05, ** *p* < 0.01 and *** *p* < 0.001 vs. blank control. Scale bar = 100 μm (applies to all panels).

To further determine the effect of octacosanol on tumours, the expression levels of proteins associated with metastasis and angiogenesis were examined in tumour tissues using IHC. IHC assay for CD31, a vascular endothelial cell surface marker in tumour tissues, showed that the percentage of positive cells was significantly lower in the octacosanol group, suggesting that octacosanol could effectively inhibit neoangiogenesis (Figure 9). In addition, the percentage of p-AKT, MMP2, MMP9 and VEGF-positive cells was significantly decreased in the octacosanol group compared with the control group (Figure 9). In contrast, the percentage of p-VEGFR2-positive cells was lower in all of them, and there was no significant difference among the three groups. In conclusion, the results of mouse experiments showed that octacosanol had a weak inhibitory ability on the proliferation of tumour cells in vivo, but could inhibit the invasive ability of tumour cells by regulating the expression of MMP2 and MMP9; meanwhile, it could effectively inhibit the expression of VEGF and its mediated neovascularisation of tumours.

## 4. Discussion

Natural compounds can disrupt tumorigenesis, progression, and metastasis by modulating diverse pathways while exhibiting minimal toxicity to normal host cells, thereby facilitating long-term administration. This study provided evidence supporting the anticancer properties of octacosanol through the inhibition of tumor metastasis and angiogenesis. Among the tumour-derived factors involved in metastasis, MMPs are most closely associated with cancer progression. MMPs play a key role in promoting tumour cell invasion by degrading various components of the extracellular environment [19]. In numerous tumors, these MMPs participate in epithelial–mesenchymal transition (EMT), invasion and metastasis, angiogenesis, and their expression is regulated by the MAPK, PI3K/AKT, and JAK/STAT signaling pathways [20,21]. The activation of the PI3K/AKT pathway specifically induces upregulation of MMP2 and MMP9, thereby promoting cancer cell invasion and migration [22]. Extensive research has been conducted on the therapeutic potential of targeting PI3K/AKT in the clinical setting, yielding evidence of its safety and efficacy [23]. Our study showed a correlative relationship: octacosanol treatment is associated with reduced activation of both PI3K/AKT and JAK3/STAT3 pathways, accompanied by decreased expression and enzyme activity of MMP2 and MMP9. These findings suggest that octacosanol may exert its anti-metastatic effects at least partly through modulating MMP expression, although the causal involvement of the upstream pathways requires further validation using specific inhibitors or genetic approaches (e.g., siRNA or CRISPR).

Hypoxia-inducible factor (particularly HIF-1α) serves as a pivotal hub linking hypoxic microenvironments to malignant tumor progression. Hypoxia is prevalent in most solid tumors, where HIF-1α and its downstream factors are frequently overexpressed, correlating closely with poor clinical outcomes [24]. During tumour development, rapid growth leads to diffusion-restricted oxygen and nutrient supply as tumour cells move away from blood vessels [25]. HIF-1 promotes ECM remodelling by regulating various MMPs, including MMP2, MMP9 and MT1-MMP [26,27], while also inducing angiogenic conversion through upregulation of pro-angiogenic factors such as VEGF, which recruits endothelial cells and constructs new blood vessels [28]. Additionally, HIF-1 expression induces immune cell recruitment, contributing to high MMP9 concentrations in the ECM [25]. The HIF-1-induced upregulation of VEGF and elevated MMP9 levels concomitantly increase soluble VEGF concentrations in tumour tissues, driving both angiogenesis and invasiveness [29]. Neovascularisation plays a dual role in promoting tumour growth by ensuring adequate nutrition supply while also serving as the main channel for tumour cell intravasation and distant metastasis [28]. Studies have shown that in cervical cancer, ginseng glucosyl oleanolate inhibits cancer cell proliferation and angiogenesis. These effects are associated with interference with the VEGF/VEGFR2 paracrine axis and the blockade of the PI3K/AKT/HIF-1α signaling pathway downstream of the autocrine axis [30]. In hypoxic microenvironments, activated MAPK/ERK promotes tumor progression by activating HIF-1 [29]. Our study demonstrates that octacosanol treatment correlates with reduced HIF-1α accumulation and decreased activation of PI3K/AKT and MAPK/ERK signaling pathways under hypoxic conditions, along with lower VEGF secretion. While these correlative data suggest a potential link, direct evidence that octacosanol blocks these pathways to inhibit HIF-1α accumulation awaits functional validation. Notably, octacosanol also effectively suppresses MMP2 and MMP9 expression under hypoxic conditions.

The regulation of vascular endothelial cell signaling plays a crucial role in angiogenesis. VEGF binding to VEGFR2 triggers receptor phosphorylation and activates downstream signaling cascades [31]. VEGF-activated VEGFR2 promotes endothelial cell proliferation through MAPK signaling via Shc and PI3K/AKT signaling via Gab1/Gab2 [32]. VEGF/VEGFR2 binding can also activate phospholipase C gamma (PLCγ) through the PI3K/AKT pathway, modulating intracellular calcium levels and enhancing eNOS production to augment vascular permeability [33,34]. Furthermore, VEGF regulates the expression of angiogenic markers including VE-cadherin, MMP2 and MMP9 [34]. Our findings show that octacosanol exhibits remarkable anti-angiogenic properties by hindering VEGFR2 activation in VEGF-stimulated HUVECs, subsequently suppressing the phosphorylation of AKT, ERK and eNOS. Additionally, octacosanol dose-dependently curtails MMP2 and MMP9 expression in HUVECs, indicating that it impedes tumor angiogenesis by targeting the VEGF-VEGFR2 interaction.

Although the inhibitory effect of octacosanol on primary tumour proliferation did not reach statistical significance in our in vivo study, which is likely attributable to the relatively small sample size and high inter-individual variability in xenograft growth, it significantly downregulated MMP2 and MMP9 expression in tumor tissues, supporting its potential to suppress tumor metastatic capacity. Meanwhile, microvessel density was markedly reduced in tumour tissues, accompanied by significantly decreased expression of VEGF and the vascular endothelial marker CD31, further confirming octacosanol’s inhibitory effect on tumour angiogenesis. We acknowledge a limitation of this study: the conclusions regarding the involvement of JAK3/STAT3 and PI3K/AKT pathways are based solely on observed changes in phosphorylation levels, without functional validation using specific inhibitors or genetic modulation. Therefore, the mechanistic claims should be interpreted as correlative evidence that supports, but does not prove, a causal role of these pathways. Future studies employing rescue experiments and targeted pathway manipulations are warranted to definitively establish the molecular mechanism underlying octacosanol’s anti-metastatic and anti-angiogenic effects.

In addition, the present study primarily employed the A549 lung adenocarcinoma cell line, a well-established and widely used model for investigating lung cancer metastasis and angiogenesis. While our previous work has confirmed similar anti-metastatic and anti-angiogenic effects of octacosanol in osteosarcoma cells [15], and existing literature has demonstrated the selective toxicity of octacosanol against cancer cells, we acknowledge that additional lung cancer cell lines and normal lung epithelial cells will be included in future studies to further validate the generalizability and tumor specificity of our findings. Regarding the octacosanol concentrations used in in vitro assays, we acknowledge that the 50–120 µg/mL range is relatively high for direct physiological relevance. It should be noted that such concentrations are commonly adopted in in vitro mechanistic studies to sufficiently explore the biological activity of natural compounds, as the static in vitro cell culture system lacks the complex in vivo pharmacokinetic and tissue accumulation environment. Importantly, our nanoemulsion formulation has significantly improved the solubility and bioavailability of octacosanol, which supports its pharmacological relevance in the in vivo setting [16]. Furthermore, no obvious systemic toxicity or adverse effects were observed in our animal model during the treatment period, indicating the safety of this intervention.

## 5. Conclusions

In conclusion, this study systematically clarified the anti-metastatic and anti-angiogenic effects of octacosanol against lung cancer. We demonstrated that octacosanol significantly suppressed A549 cell invasion and migration in vitro, and this effect was associated with downregulation of MMP2/9 expression and activity, accompanied by reduced phosphorylation of the JAK3/STAT3 and PI3K/AKT pathways. It also targeted VEGF-VEGFR2 to inhibit endothelial tube formation, and reduced hypoxia-induced HIF-1α accumulation to suppress VEGF secretion and pro-angiogenic stimuli. In vivo xenograft experiments validated these effects: octacosanol markedly inhibited p-AKT, MMP2 and MMP9 expression in tumor tissues, reduced microvessel density, and downregulated VEGF and CD31, confirming its in vivo anti-metastatic and anti-angiogenic efficacy. Collectively, these findings provide initial preclinical experimental evidence supporting octacosanol as a promising adjuvant agent targeting tumor metastasis and angiogenesis, laying a theoretical basis for developing related nutraceuticals for the clinical management of lung cancer.

## Figures and Tables

**Table 1:** List of PCR primers.

Gene Name	Primer	Sequence (5′–3′)
GAPDH	Forward	CAGGAGGCATTGCTGATGAT
	Reverse	GAAGGCTGGGGCTCATTT
MMP1	Forward	AGCCATCACTTACCTTGCACTGAG
	Reverse	CCACATCAGGCACTCCACATCTG
MMP2	Forward	AGCCAAGCGGTCTAAGTCCAGAG
	Reverse	GGAATGAAGCACAGCAGGTCTCAG
MMP9	Forward	TCCTGGTGCTCCTGGTGCTG
	Reverse	CTGCCTGTCGGTGAGATTGGTTC
MMP13	Forward	AGTCATGGAGCTTGCTGCATTCTC
	Reverse	TCCTGGCTGCCTTCCTCTTCTTG
MMP14	Forward	CCTGCCTGCGTCCATCAACAC
	Reverse	GCCTCATCAAACACCCAATGCTTG

## Data Availability

The authors confirm that the data supporting the findings of this study are available within the article. All data generated or analyzed in the current study are available from the corresponding author on reasonable request.

## Abbreviations

The following abbreviations are used in this manuscript:

CAM	Chicken chorioallantoic membrane
Cox-2	Cyclooxygenase-2
ECM	Extracellular matrix
EMT	Epithelial–mesenchymal transition
FBS	Fetal bovine serum
HIF-1α	Hypoxia-inducible factor-1α
HUVECs	Human umbilical vein endothelial cells
MMPs	Matrix metalloproteinases
VEGF	Vascular endothelial growth factor
VEGFR2	Vascular endothelial growth factor receptor 2
IHC	Immunohistochemistry
NC	Negative control
PLCγ	Phospholipase C gamma
